# The Economic Impact of Artificial Intelligence in Health Care: Systematic Review

**DOI:** 10.2196/16866

**Published:** 2020-02-20

**Authors:** Justus Wolff, Josch Pauling, Andreas Keck, Jan Baumbach

**Affiliations:** 1 TUM School of Life Sciences Weihenstephan Technical University of Munich Freising Germany; 2 Strategy Institute for Digital Health Hamburg Germany

**Keywords:** telemedicine, artificial intelligence, machine learning, cost-benefit analysis

## Abstract

**Background:**

Positive economic impact is a key decision factor in making the case for or against investing in an artificial intelligence (AI) solution in the health care industry. It is most relevant for the care provider and insurer as well as for the pharmaceutical and medical technology sectors. Although the broad economic impact of digital health solutions in general has been assessed many times in literature and the benefit for patients and society has also been analyzed, the specific economic impact of AI in health care has been addressed only sporadically.

**Objective:**

This study aimed to systematically review and summarize the cost-effectiveness studies dedicated to AI in health care and to assess whether they meet the established quality criteria.

**Methods:**

In a first step, the quality criteria for economic impact studies were defined based on the established and adapted criteria schemes for cost impact assessments. In a second step, a systematic literature review based on qualitative and quantitative inclusion and exclusion criteria was conducted to identify relevant publications for an in-depth analysis of the economic impact assessment. In a final step, the quality of the identified economic impact studies was evaluated based on the defined quality criteria for cost-effectiveness studies.

**Results:**

Very few publications have thoroughly addressed the economic impact assessment, and the economic assessment quality of the reviewed publications on AI shows severe methodological deficits. Only 6 out of 66 publications could be included in the second step of the analysis based on the inclusion criteria. Out of these 6 studies, none comprised a methodologically complete cost impact analysis. There are two areas for improvement in future studies. First, the initial investment and operational costs for the AI infrastructure and service need to be included. Second, alternatives to achieve similar impact must be evaluated to provide a comprehensive comparison.

**Conclusions:**

This systematic literature analysis proved that the existing impact assessments show methodological deficits and that upcoming evaluations require more comprehensive economic analyses to enable economic decisions for or against implementing AI technology in health care.

## Introduction

### Background

In times of value-based health care and also because of the high share of the health care industry in the overall economy, economic impact assessment is of increasing importance. For instance, health care expenditures account for approximately US $3.5 trillion out of US $19.4 trillion (18%) of the overall gross domestic product (GDP) in the United States and for approximately US $0.4 trillion out of US $3.7 trillion (11.5%) of the overall GDP in Germany [1,2]. Accordingly, the cost impact of digital health applications has also been analyzed in several studies.

In 2002, in a review of cost-effectiveness studies in the context of telemedicine interventions, Whitten et al [3] revealed that only 55 out of 612 identified articles presented actual cost-benefit data, which were required to be included in a detailed review. In addition, after analyzing these articles, the authors concluded that the provided evidence was not sufficient to assess whether telemedicine represents a cost-effective mean of delivering health care [3].

More than a decade later, in 2014, Elbert et al [4] described in a review of systematic reviews and meta-analyses regarding electronic health (eHealth) interventions in somatic diseases that out of 31 reviews, 7 papers concluded that digital health is effective or cost-effective, 13 underlined that evidence is promising, and the other 11 found only limited or inconsistent proof. They also highlighted that the development and evaluation of strategies to implement effective or cost-effective eHealth initiatives in daily practice needed to be significantly enhanced [4].

In another systematic review study on the economic evaluations of eHealth technologies from 2018, Sanyal et al [5] analyzed multiple databases with publications between 2010 and 2016. On the basis of 11 studies that fulfilled the inclusion criteria, the authors found that most of the studies demonstrated efficacy and cost-effectiveness of an intervention using a randomized control trial and statistical modeling. However, there was insufficient information provided on the feasibility of adopting these modeling technologies. Thus, the paper emphasizes that the current level of evidence is inconclusive and that more research is needed to evaluate possible long-term cost benefits [5].

Research in this segment has been continuously intensified, and in several studies, the digital health cost-effectiveness, for example, of telemedicine for remote orthopedic consultations [6], digital behavioral interventions for type 2 diabetes and hypertension [7], and internet-based interventions for mental health [8] was analyzed in detail.

As significant medical quality enhancements and cost-saving improvements through artificial intelligence (AI) as one of the key emerging technologies in digital health are expected, the economic impact assessment of AI in health care has a crucial role for all stakeholders in health care and, thus, needs to be analyzed in detail.

### Objective

It was systematically investigated whether the existing cost-effectiveness evaluations meet the established quality criteria to enable comprehensive decision making regarding the implementation of AI in health care. On the basis of these thorough economic assessments, the necessary information to decide for or against the application of AI in hospitals, industry, and payer context will be provided.

## Methods

A systematic literature review was performed as described in the following sections.

### Search Strategies

A literature search was conducted utilizing the PubMed database and using the search terms provided in Table 1.

**Table 1:** Search terms (title and abstract) in the PubMed analysis (conducted on July 29, 2019).

Components	Syntax	Hits, n
Artificial intelligence OR machine learning AND cost effectiveness	(Artificial intelligence [title/abstract] OR machine learning [title/abstract]) AND cost effectiveness [title/abstract]	54
Artificial intelligence OR machine learning AND economic impact	(Artificial intelligence [title/abstract] OR machine learning [title/abstract]) AND economic impact [title/abstract]	9
Artificial intelligence OR machine learning AND cost saving	(Artificial intelligence [title/abstract] OR machine learning [title/abstract]) AND cost saving [title/abstract]	3

The search terms *Artificial Intelligence* and *Machine Learning* for the overall segment are not exhaustive as eg, *Decision trees*, *Support vector machines*, or *Deep neural networks* could also have been used as search terms for the database queries. Nonetheless, as strategic decisions based on economic impact are mostly made on a strategic managerial and medical level without a specific technological background, the most frequently used search terms regarding AI in health care have been used. In addition, it is highly probable that papers about, for example, *deep neural networks* would also include such terms as *artificial intelligence*, *support vector machines*, and *machine learning* at least in the abstract. Finally, it was decided to use a Google Trends analysis comparing the most frequently used search terms regarding AI in health care over the last 12 months globally [9]: The terms *Artificial Intelligence* and *Machine Learning* have been used the most by far, as illustrated in Multimedia Appendix 1.

### Inclusion Criteria

For the publications identified through the PubMed searches, the titles, abstracts, and full texts have been reviewed. Publications were included into the subsequent analysis if they were (1) published journal articles, (2) written in English language, and (3) published no more than 5 years ago. With regard to the content, the publications were included if they focused on at least one of the following content sectors: (1) a comprehensive description of an AI functionality, (2) an evaluation of the economic efficiency and outcomes of the AI functionality, and (3) quantitative outcomes of the AI functionality in at least one health care system. Furthermore, only publications describing concrete medical and economic outcomes, such as cost savings per patient per year, and reviews or meta-analyses comparing AI solutions have been included.

### Exclusion Criteria

Exclusion criteria for an article were defined as follows: (1) the title did not cover a topic related to AI in health care; (2) neither the title nor the abstract contained a description of an AI application in health care; or (3) the title, abstract, or full text did not elaborate on the quantitative economic outcome of AI in health care application in any health care system. In contrast to other previous research review approaches, such as those chosen by Elbert et al [4] or Ekeland et al [10], the third exclusion criterion was covered. Although this significantly limited the number of cost-effectiveness studies included, it was applied to compare the different cost-effectiveness analysis approaches and not only the health- or process-related outcomes without quantified economic impact from a national or international health care perspective.

**Figure 1:**
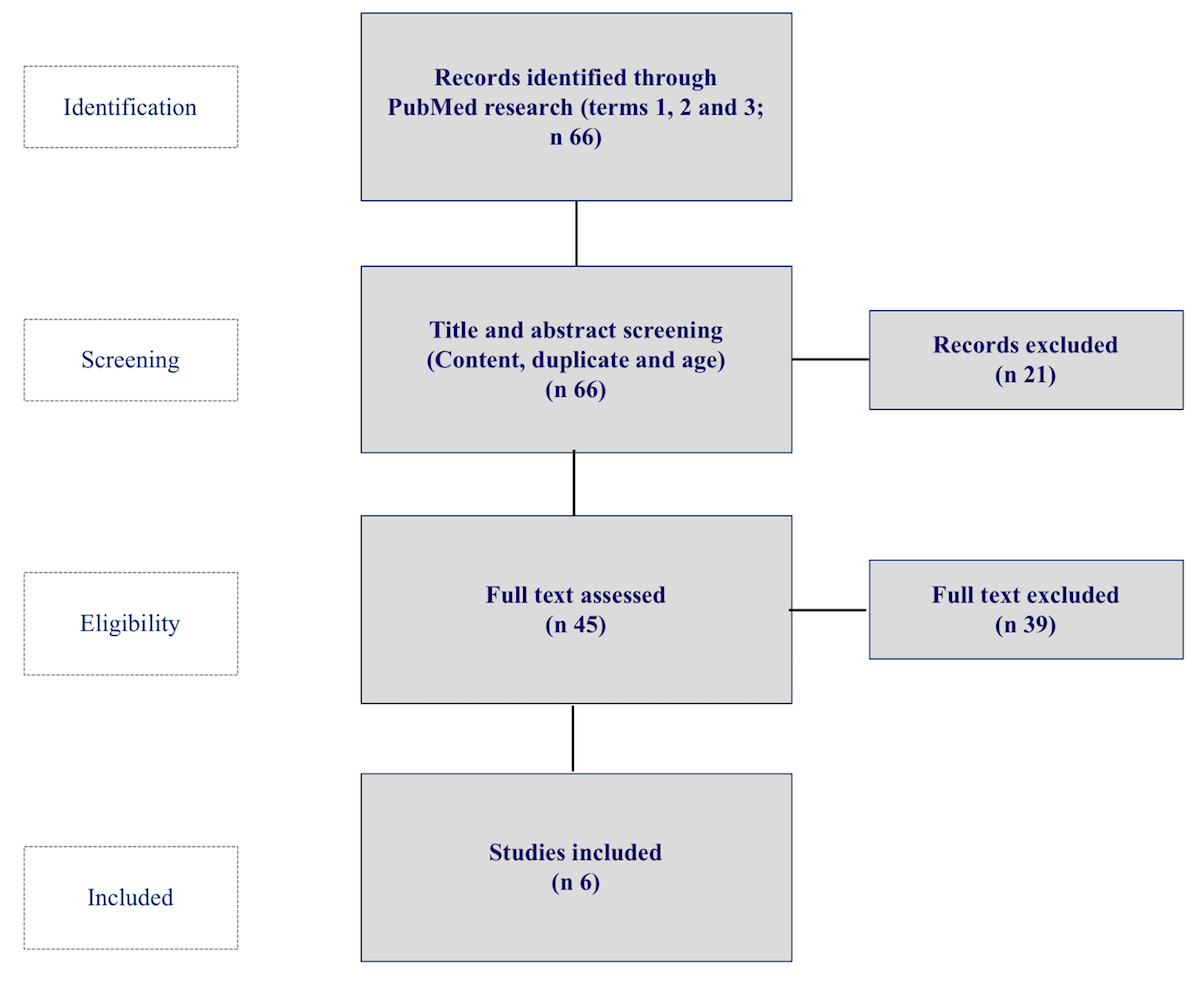
Study selection and identification flowchart.

After identifying potential studies for inclusion via the PubMed search, as previously described, the evaluation took place in two steps (Figure 1). First, all titles, abstracts, and full texts were screened for the fulfillment of the inclusion and exclusion criteria. Second, publications viable for inclusion were assessed with a quality criteria catalog, which is explained in section Quality Criteria for Economic Impact Assessment.

### Quality Criteria for Economic Impact Assessment

A combined criteria catalog for cost-effectiveness studies was designed. Besides own criteria, additional evaluation aspects from classical health care effectiveness studies and digital health assessments were considered [5,11]. The quality criteria are summarized in Table 2.

**Table 2:** Quality criteria for economic impact assessment.

Criteria	Explanation	Source
Description of cost-effectiveness of AI^a^ solution	Level of detail of cost-effectiveness explanation	Authors
Hypothesis formulation	Analysis if a comprehensive question has been formulated that allows AI cost-effectiveness evaluation (eg, comparing the AI approach with the recommended guideline routine)	Study by Haycox and Walley [11]
Cost-effectiveness perspective	Impact of change in the cost of stand-alone functionality vs overall reduction of burden of care	Study by Haycox and Walley [11]
Consideration of cost alternative	Analysis if the cost-saving results could also have been achieved with an alternative strategy	Study by Haycox and Walley [11]
Benefit today	Net present value of the AI service, including upfront investments and running costs	Study by Haycox and Walley [11]
Verification of base case	Analysis of cost-effectiveness of the AI solution based on benchmarking with base case data	Study by Sanyal et al [5]

^a^AI: artificial intelligence.

## Results

### Quality Criteria Evaluation

Quality criteria have been applied to assess the economic impact assessments on a scale of 1 to 3 (1=superficial coverage, 2=solid coverage, and 3=detailed explanation). As outlined above, 6 publications have been assessed regarding the described quality criteria for economic impact evaluation. An overview of the analysis of the publications [12-17] is given in Multimedia Appendix 2.

### Quality Assessment Results

We first conclude that the level of detail of description of the cost-effectiveness measurement was overall high as the descriptions were for the most part precise and detailed, for instance, “for an incremental cost effectiveness threshold of €25,000/quality-adjusted life year, it was demonstrated that the AI tool would have led to slightly worse outcomes (1.98%), but with decreased cost (5.42%)” [14]. Overall, 5 out of the 6 publications had a very high level of detail, and only 1 study had a medium level of detail in the general description (only a positive/negative cost-saving impact description and no further outcome explanations have been provided [13]).

Second, the hypothesis formulation (eg, cost saving through machine learning–based prediction models to identify optimal heart failure patients for disease management programs to avoid 30-day readmissions [17]) was clear and accurate across all publications. All comprised well-explained and coherent hypothesis formulations.

Third, the cost-effectiveness perspective had in all cases a *health care system* context, although additional perspectives could have been included, such as ambulant or nurse perspectives. Furthermore, 5 studies demonstrated a comprehensive health care system perspective, whereas 1 could have been extended from a hospital to an overall system view [13].

Fourth, the cost alternative consideration, that is, the analysis of whether the cost-saving results could also have been achieved alternatively, was mostly missing. Only 2 papers elaborated on the different alternatives in detail, for example, differentiating on the levels of risks of the respective patient groups or different treatment options. Besides these 2 publications [12,16] that covered various alternatives to achieve a similar cost saving, the remaining 4 publications did not elaborate on such cost alternative considerations at all.

Fifth, the benefit achieved today, that is, in terms of a net present value (NPV) including not only the benefits but also the necessary investment for the AI implementation and the operational costs of an AI service delivery, was not covered in any of the 6 studies. Only 1 study compared AI vs non-AI scenarios but without providing a NPV calculation. Hence, all 6 studies included a quantification of economic outcomes but failed to calculate an overall NPV.

Finally, the verification of the base case was conducted using different approaches across the 6 studies. Mostly solid data sources have been collected in dedicated AI-focused studies based on, for example, comparison of cost with/without the algorithm, reimbursement code analysis, or benchmarking of the result with the reported performance of other clinics. All papers presented a cost-effectiveness measurement based on a comprehensive comparison dataset.

One additional aspect that emerged throughout the analysis was the measurement of resource usage, which was (almost) in all papers conducted via a top-down approach, meaning from an overall health care perspective but not from a single cost split per task. In this way, important cost drivers of potentially *hidden* stakeholders could have been missed (eg, additional workload for ambulatory care if a hospital treatment is altered).

## Discussion

### Principal Findings

Overall, the outcomes of the analysis described above can be split into two result categories, namely, general feedback from the analysis and detailed assessment of the studies that have been included in the review process based on the study’s inclusion and exclusion criteria.

Generally, only a few publications can be found for the economic impact assessment of AI in health care. On the basis of the different search terms that include the most frequently searched phrases by far in this segment (*Artificial Intelligence* and *Machine Learning*) in combination with the economic impact (*Cost effectiveness*, *Economic impact*, *Cost saving*), there were only 66 PubMed hits. As AI strategies and consequent decision-making processes depend on solid data as the basis for decision making, this is a significant challenge for both the management and medical staff, for example, when general pro and contra decisions and specific implementations regarding AI are discussed.

When accounting for the details given in the identified AI in health care publications, the economic assessment quality shows several deficits that need to be overcome in the future. Only 6 out of the 66 publications (9%) could be included in the detailed assessment. Out of these 6 studies, none comprised a complete cost-benefit analysis; rather, they all focused on fragmented cost or cost-saving aspects.

**Figure 2:**
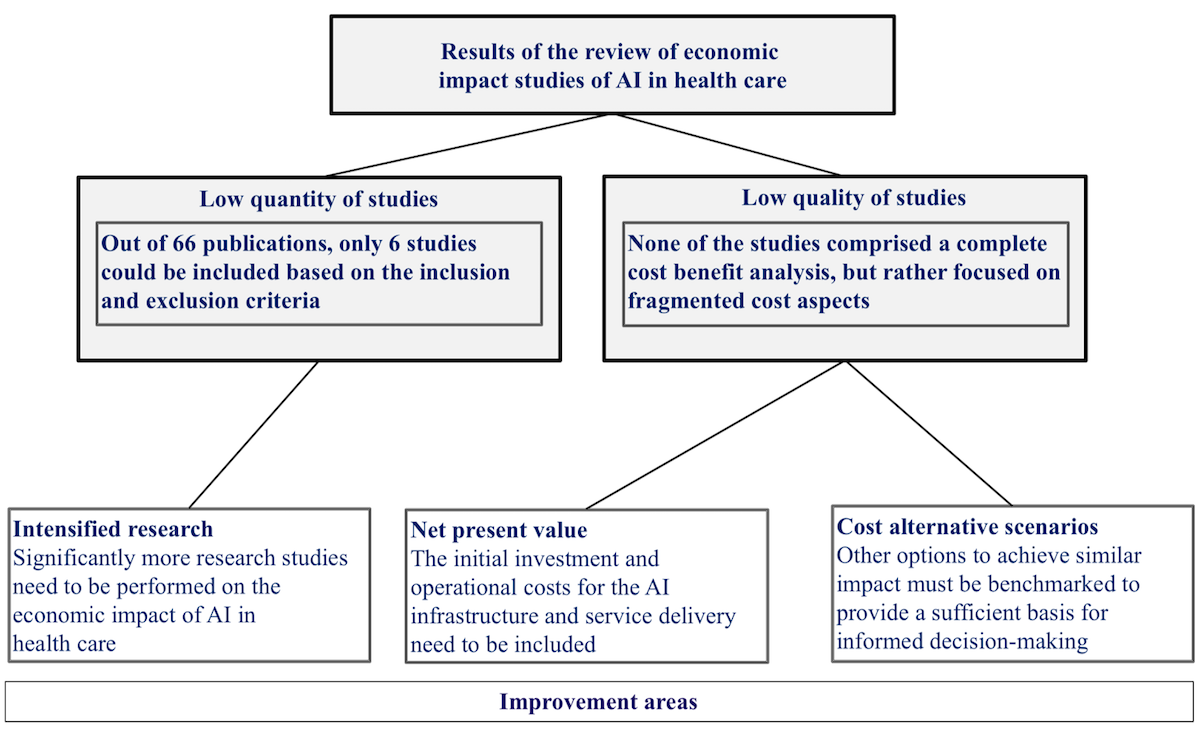
Result of the literature review and improvement areas for economic impact assessment of artificial intelligence (AI) in health care.

Room for improvement (Figure 2) has been identified in two main areas:

- First, initial investment and operational costs for the AI infrastructure and service need to be included in the assessment. This is a core element for any strategic decision-making process, and the complete initial and operational investment costs for an AI solution must be compared with the expected economic benefits to provide concrete decision-making support.
- Second, further options to achieve similar impact must be evaluated to reach a sufficient basis for comprehensive and transparent decision-making, allowing comparisons among different strategic and investment options (eg, a genetic sequencing process or different medical expertise allocation for a diagnosis and treatment outcome improvement could also be applied instead of an AI-driven patient screening).

The conducted review has a rather narrow focus on economics and business perspectives of AI in health care. However, the literature review revealed further significant success factors for AI, for example, regarding the legal framework, such as compliance with data security, protection, and privacy policies, and also universally accepted technological requirements to enable comprehensive data collection and to analyze content while complying with data privacy requirements. Despite the benefits in assisting diagnostic and therapeutic decisions, so far, no standards for these legal and technological issues have been defined, and these aspects should be analyzed in future research with a broader focus.

Furthermore, aside from the sole economic quantitative aspects, the qualitative aspects of AI in health care for patients and the society require further research. For instance, in rural areas where the availability of primary care physicians is limited, AI can replace processes through focused test support, for example, for type 2 diabetes, thus addressing the challenges of demographic change [18]. The comparison between AI and physicians with regard to diagnosis performance demonstrated that AI can deliver equal results, for example, in image recognition–related fields [19]. This can, among others, also support a reallocation of medical capacities. In addition, AI can also enable a shift from a generalized to a more personalized treatment. AI-steered outcome prediction and clinical decision support processes are already used today, for instance, for patients in radiation therapy [20].

Prior reviews in the digital health segment categorized the results into groups, for example, computerized decision support system, Web-based physical activity intervention, internet-delivered cognitive behavioral therapy, and telehealth. In addition, user’s age was differentiated (eg, children vs old patients), and shortcomings such as a missing difference between short- and long-term cost savings were highlighted [5]. They also covered challenges that go beyond the cost-effectiveness aspect and mentioned, for instance, that the way to implement digital health in daily practice is still unclear [4] or that patient perspectives and collaborative approaches among a variety of stakeholders are needed [10].

Note that the focus on AI in health care required considering novel factors and a refined search strategy as compared with typical reviews on digital health resulting in differential results. First, in contrast to other reviews, Google Trends has proven to be an effective tool to narrow the search space for a representative collection of results. On the basis of the Google Trends analysis, the key phrases *Artificial Intelligence* and *Machine Learning* could be identified as the most frequently used terms by far. Second, the review covered a higher percentage of included studies after applying the defined inclusion and exclusion criteria (9% of the analyzed papers were included), whereas prior reviews had much lower inclusion rates—8% (55/612) in the study by Whitten et al [3], 2% (31/1657) in the study by Elbert et al [4], or 0.1% (11/1625) in the study by Sanyal et al [5]). This was because of two reasons: (1) AI as a subsegment of digital health in business and industry is still not covered well in scientific publications and (2) the high importance of quantitatively reported outcomes required as inclusion criterion. Third, the evaluation of cost-effectiveness studies has been conducted with a quality criteria catalog from a management perspective. As AI implementation is cost- and labor-intensive and decisions are not exclusively driven by medical improvement rates, the business management decision making basis has been chosen as crucial for positive implementation decisions and subsequent widescale applications. The addition of the business management view includes classical cost factors (onetime and running expenses) as well as decisions among different strategies to deliver cutting edge health services.

### Conclusions

Current research covers impact assessments of AI in health care rather moderately and shows qualitative deficits in methodology. Future cost-effectiveness analyses need to increase in number and quality. They should include initial investment and running costs as well as the comparison with alternative technologies. This way a comprehensive and clearly segmented cost-benefit evaluation can be provided, which will serve as a sufficient basis for decision making regarding AI implementations.

## Appendix

Multimedia Appendix 1 Screenshot of a Google Trends analysis of search terms related to artificial intelligence in health care globally over the last 12 months (conducted on October 9, 2019).

Multimedia Appendix 2 Analysis of the included economic impact studies.

## Abbreviations

AI: artificial intelligence
eHealth: electronic health
GDP: gross domestic product
NPV: net present value
