# Supplementary figures and images for: The Economic Impact of Artificial Intelligence in Health Care: Systematic Review
Source: J Med Internet Res. 2020 Feb 20;22(2):e16866. doi: 10.2196/16866 (PMC7059082; doi:10.2196/16866)

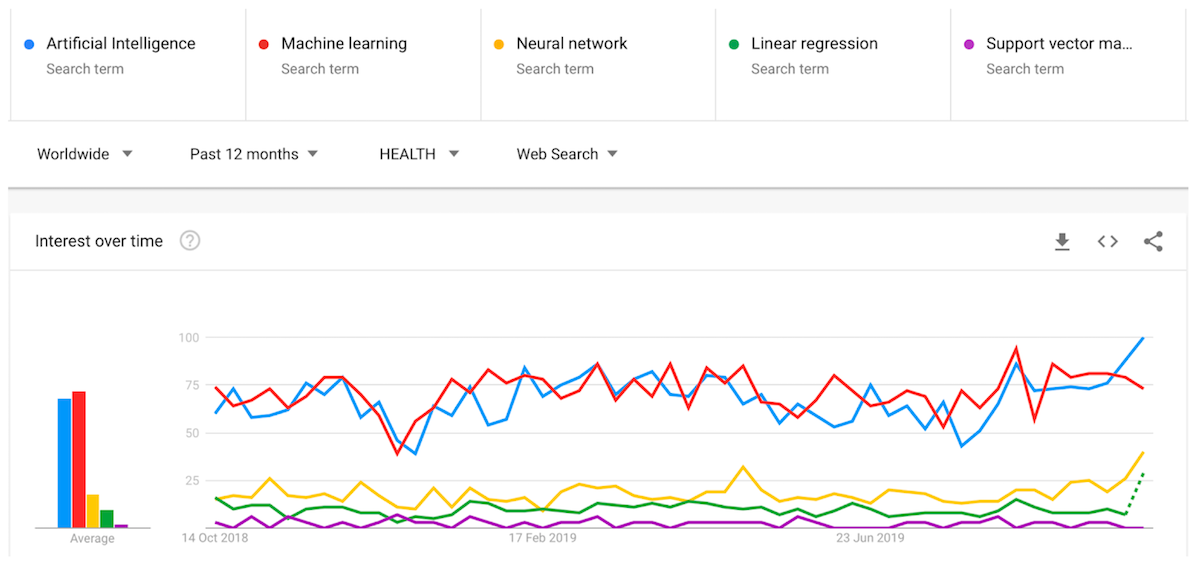

Supplement: Multimedia Appendix 1 [file jmir_v22i2e16866_app1.png]

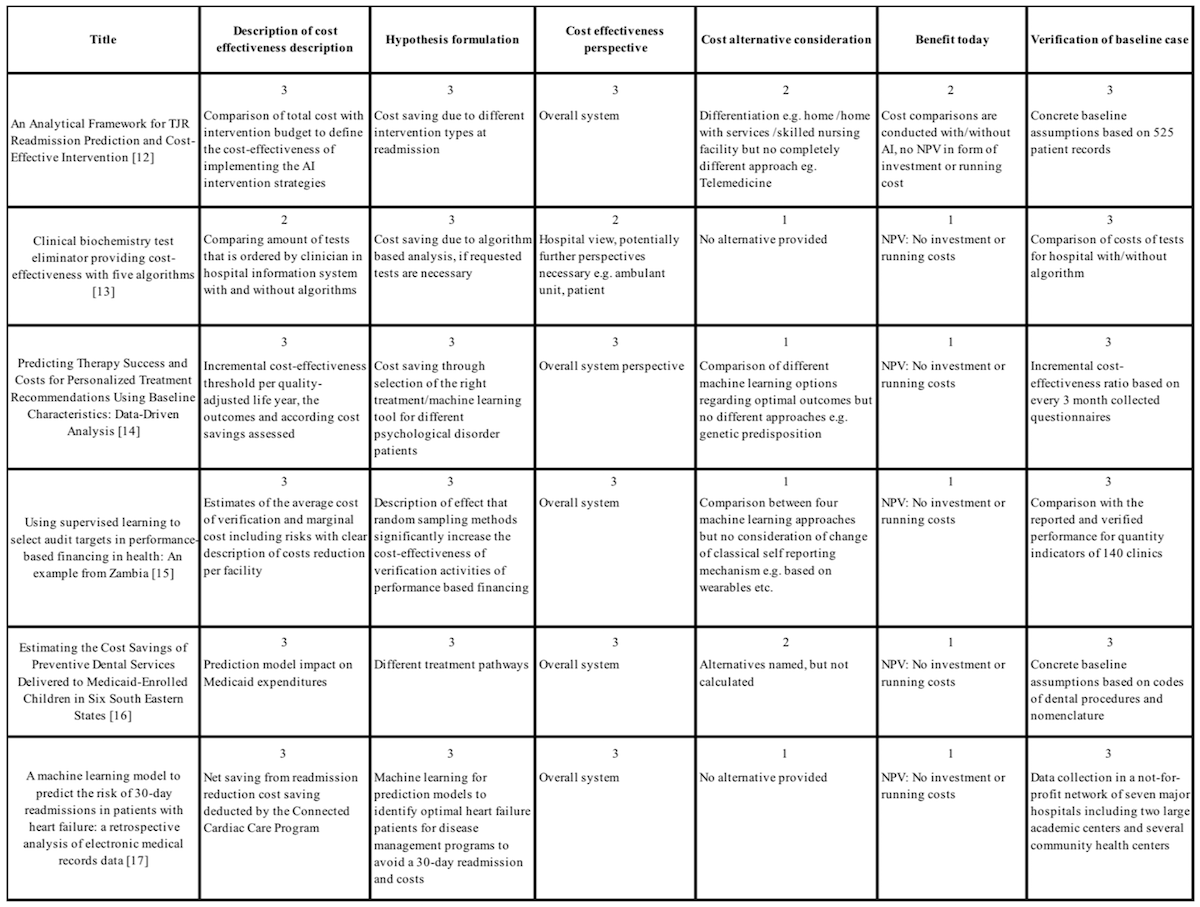

Supplement: Multimedia Appendix 2 [file jmir_v22i2e16866_app2.png]
